# Antihyperglycemic Potential of *Spondias mangifera* Fruits via Inhibition of 11β-HSD Type 1 Enzyme: In Silico and In Vivo Approach

**DOI:** 10.3390/jcm12062152

**Published:** 2023-03-09

**Authors:** Shadma Wahab, Mohammad Khalid, Mohammed H. Alqarni, Mohamed Fadul A. Elagib, Ghadah Khaled Bahamdan, Ahmed I. Foudah, Tariq M. Aljarba, Mons S. Mohamed, Nazik Salih Mohamed, Muhammad Arif

**Affiliations:** 1 Department of Pharmacognosy, College of Pharmacy, King Khalid University, Abha 62529, Saudi Arabia; 2 Department of Pharmacognosy, College of Pharmacy, Prince Sattam Bin Abdulaziz University, P.O. Box 173, Al-Kharj 11942, Saudi Arabia; 3 Department of Periodontics and Community Dental Sciences, College of Dentistry, King Khalid University, Abha 61421, Saudi Arabia; 4 Department of Pharmacognosy, Faculty of Pharmacy, University of Khartoum, Khartoum 11111, Sudan; 5 Department of Pharmacognosy, Imperial University College, Khartoum 11111, Sudan; 6 Department of Pharmacognosy, Faculty of Pharmacy, Integral University, Lucknow 226026, India

**Keywords:** natural products, hypoglycemia, *Spondias mangifera*, anti-hyperglycemic, 11β-HSD1, in silico, in vitro, in vivo

## Abstract

The 11 β- hydroxysteroid dehydrogenase 1 (11 β-HSD1) is hypothesized to play a role in the pathogenesis of type 2 diabetes and its related complications. Because high glucocorticoid levels are a risk factor for metabolic disorders, 11β-HSD1 might be a viable therapeutic target. In this investigation, docking experiments were performed on the main constituents of *Spondias mangifera* (SM) oleanolic acid, β-amyrin, and β-sitosterol to ascertain their affinity and binding interaction in the human 11β-hydroxysteroid dehydrogenase-1 enzyme’s active region. The results of in vitro 11β HSD1 inhibitory assay demonstrated that the extract of *S. mangifera* had a significant (*p* < 0.05) decrease in the 11-HSD1% inhibition (63.97%) in comparison to STZ (31.79%). Additionally, a non-insulin-dependent diabetic mice model was used to examine the sub-acute anti-hyperlipidemic and anti-diabetic effects of SM fruits. Results revealed that, in comparison to the diabetic control group, SM fruit extract (SMFE) extract at doses of 200 and 400 mg/kg body weight considerably (*p* < 0.05 and *p* < 0.01) lowered blood glucose levels at 21 and 28 days, as well as significantly decreased total cholesterol (TC) and triglycerides (TG) and enhanced the levels of high-density lipoprotein (HDL). After 120 and 180 s of receiving 200 and 400 mg/kg SMFE, respectively, disease control mice showed significantly poorer blood glucose tolerance (*p* < 0.05 and *p* < 0.01). SMFE extract 200 (*p* < 0.05), SMFE extract 400 (*p* < 0.01), and Glibenclamide at a dosage of 5 mg/kg body weight all resulted in statistically significant weight increase (*p* < 0.01) when compared to the diabetic control group after 28 days of treatment. According to in silico, in vitro, and in vivo validation, SMFE is a prospective medication with anti-diabetic and hypoglycemic effects.

## 1. Introduction

Herbal drugs have a promising impact on the human health care system globally. More than 80% of the world’s population uses herbal remedies to treat a wide range of potentially fatal acute and chronic conditions, as documented in the WHO database [1]. There are over 800 different medicinal plants that have been identified as having anti-diabetic properties, but no herbal drug has been shown to have side effects or toxicities compared to modern synthetic drugs [2]. The incidence of chronic degenerative illnesses such as type 2 diabetes has been a direct result of poor food habits and sedentary lifestyles [3]. Elevated blood glucose levels are a hallmark of diabetes mellitus, which includes a variety of biological processes, including insulin secretion, insulin resistance, and glucose absorption [4]. IFD data show that around 425 million people worldwide possess type 2 diabetes. In diabetic patients, the most common diagnostic symptoms are neuropathy, retinopathy, cardiovascular disease, skin complications, and nephropathy [5]. Most of the proteins involved as being essential in the progression of type 2 diabetes include glucokinase, insulin receptor substrate, 11-hydroxysteroid dehydrogenase, dipeptidyl peptidase IV, interleukin 1 beta, AMP-activated protein kinase, peroxisome proliferator-activated receptor gamma, C-reactive protein, glutamine fructose-6-phosphate amidotransferase, tyrosine kinase insulin receptor, insulin receptor, protein tyrosine phosphatases, and protein kinase B [6].

Alterations to one’s diet, physical activity, and anti-diabetic and anti-obesity drugs are some of the several medical methods that have been created to combat type 2 diabetes. Sulfonylureas, thiazolidinediones, metformin, and insulin are some oral hypoglycemic agents used to treat diabetes. However, these drugs have side effects such as lactic acidosis, weight gain, hyperglycemia, stomach pain, diarrhea, anorexia, flatulence, and sleepiness, along with other problems such as failure to control blood sugar and different drug responses in other people [7,8]. Therefore, new therapeutic targets and anti-diabetic agents with fewer side effects and improved effectiveness are needed to prevent and manage T2DM. Researchers worldwide study the bioactive phytoconstituents found in herbal plants to treat various diseases because herbal medicines do not cause life-threatening side effects [9].

*Spondias mangifera* (Anacardiaceae) is a small woody plant known as Amra or wild mango tree. It is also known as amrata in Ayurvedic medicine. Amra is grown in many parts of India, including Odisha, Maharashtra, and Punjab. Every part of the plant is employed to treat different illnesses. It is well known for its medicinal and nutritional supplement properties [10]. Fruits are edible in Assam and West Bengal. It is an effective treatment for a wide range of diseases, including diarrhea, dysentery, and nausea, as well as bilious dyspepsia. Fruits and vegetables are also beneficial in treating muscular and anti-inflammatory pain. The bark of the plants is utilized in the treatment of gonorrhea, and the roots are employed in the treatment of menstrual problems [11]. Tribal women in Malaysia and India used a hot aqueous extract of the root internally to regulate menstrual irregularities, and its paste was applied externally as a massage to relieve muscular pain [12]. The aerial parts of the plant contain daucosterol, 24-methylene, sitosterol, stigmast-4-en-3-one, cycloartanone, and lignoceric acid. In addition to that, the fruits contain β-amyrin as well as oleanolic acid [13]. The active compounds in medicinal plants may affect several targets through numerous processes. As a result, they offer a lot of potential and benefits in treating complicated diseases such as diabetes and its consequences [14].

Inhibitors of the enzyme 11-β-HSD1 are one of the more recent classes of compounds being developed to combat the problems of diabetes. Based on several pieces of research evidence, it has been suggested that 11-β-HSD1-mediated intracellular cortisol production subsidizes the progress of T2DM and its accompanying complications [15]. Carbenoxolone (CBO) is one of the most widely used 11-β-HSD1 inhibitors. It is a synthetic analog of 18-glycyrrethinic acid, a type of triterpene that is present in many plants [16]. Until recently, several potent and selective 11-β-HSD1 inhibitors had been described, and a number of these compounds are currently undergoing clinical trials [17].

Consequently, exploring the seemingly inexhaustible phytoconstituents resource for selective 11-β-HSD1 inhibitors will be a fruitful endeavor. Computational molecular modeling has rapidly emerged as a fundamental strategy for discovering and creating new pharmaceuticals based on natural molecules. In silico drug design methods help speed up the process of creating pharmaceuticals from plants. Additionally, computer modeling has shown how the interactions between disease-related target macromolecules and drug-like substances work at the molecular level [18]. Thus, with the flexibility and dynamic properties of the human 11β-hydroxysteroid dehydrogenase-1 enzyme in mind, the present study evaluated the potential of underused, green, raw, edible fruits of *S. mangifera* collected from the eastern part of India as an anti-hyperglycemic drug based on its ability to restrain the 11-β-HSD1 pathway via in silico prediction studies. In addition, the docking simulation results and in vitro 11β HSD1 inhibitory activity were verified by validating the sub-acute anti-diabetic and anti-hyperlipidemic effects in a mice model.

## 2. Materials and Methods

### 2.1. Plant Collection and Authentication

*S. mangifera* fruit for this research was bought from a market in Lucknow, India. The plant was authenticated by Dr. Muhammad Arif, Department of Pharmacognosy Faculty of Pharmacy, Integral University, Lucknow, India. The herbarium with a voucher specimen number IU/PHAR/HRB/20/16 has been deposited for future reference.

### 2.2. Chemicals

This study’s chemicals, drugs, and solvents were of the highest analytical quality. Thermo Fisher Scientific, Waltham, MA, USA, supplied the active material for Glibenclamide and Streptozotocin. Methanol was purchased from the Val-de-Reuil, France, and Calro Erba Reagents. Methyl ethyl acetate was purchased from the Labort Fine Chemical Private Limited, Surat, Gujrat, India. Test strips for an Accu-Check^®^ Active glucometer were used during the experiment. 3,5-dinitrosalicylic acid (DNSA), a glucometer, was bought from Sigma-Aldrich Company in St. Louis, Missouri, in the United States. 

### 2.3. Preparation of Coordinate Files 

Carbenoxolone’s X-ray crystal structures and PDB ID: 2ILT’s crystal structures were obtained from the Protein Data Bank (https://www.rscb.org/pdb, accessed on 11 March 2021). All heteroatoms and water molecules in the obtained target files were eliminated in order to create the protein structure. Target structures were further optimized, and energy was minimized using the Swiss PDB viewer V.4.1.0 program [19].

### 2.4. Preparation of Ligands

The PubChem database (https://pubchem.ncbi.nlm.nih.gov/, accessed on 11 March 2021) was used to download the 3D SDF structures of chemicals and standard medications (carbenoxolone and PDB ID: 2ILT), which were then translated to PDB format using Open Babel. Using the graphical user interface of PyRx virtual screening tool, Python prescription 0.8, energy was reduced and transferred to PDBQT format. 

### 2.5. Molecular Docking

The principal bioactive constituent of (*S. mangifera*) was chosen for molecular docking studies to understand better prospective molecular interactions based on their affinity to interact with distinct target enzymes. Auto-Dock 4.0 docking experiments were carried out on Windows 10 (×86) PCs. Python 2.7-based MGL tools 1.5.6 were part of the software package. Using Auto Grid, precalculated grid maps of the interaction energies of different atom types were created. A grid box with a grid map of 60 × 60 × 60 points and a grid spacing of 0.375 Å was built for each docking. From the UniProt database, we determined the precise location of the ligand binding site in each protein, which served as the focal point for the grid maps [20]. Docking was accomplished using a Lamarckian genetic algorithm (LGA) with the default settings. All the computations were carried out on Cygwin and were used to generate the grid parameter file (gpf file) and a docking parameter file (dpf file) for each ligand. Based on the binding energy, the docked conformations of each ligand were grouped into clusters, and the top-ranked clusters were used for further research. The position that had the lowest binding energy was deemed to be the most suitable one, and ligand–receptor interactions were further examined in this pose. 

### 2.6. Analysis and Visualization

The docked molecules with poses showing the lowest binding energies within the active site were visualized using Discovery Studio Visualizer Client (Windows 64-bit) (267 MB).

### 2.7. Preparation of S. mangifera Fruit Extract

For the preparation of *S. mangifera* fruit extract, at first, each fruit was divided into four pieces, and then it was dried in the open air at room temperature. To ensure a steady weight, the dried fruits were further diced using the chopper and dried at 40–45 °C in an oven for another 2–3 days. The grinder was then used to reduce the dried materials that had been acquired to a coarse powder. First, the defatting of the air-dried fruits powder was performed with petroleum ether (40–60 °C), and then the defatted marc was macerated for 72 h in 80% ethanol to ensure complete extraction. It was filtered using cotton and then re-extracted to exhaust the bioactive constituents of the fruits thoroughly. After the second filtration, both the filtrates were combined and concentrated to get a sticky mass of *S. mangifera* fruit extract (SMFE) under reduced pressure on a rotatory evaporator at a temperature of 40 °C [21].

### 2.8. HPLC Analysis of Oleanolic Acid

The analysis was performed using an RP-HPLC system equipped with a PDA detector (e2695 Separation module, manufactured by Waters, Milford, MA, USA). In order to conduct the analysis, a high-performance liquid chromatography (HPLC) system that was preheated to 30 °C and fitted with an RP-C18 column manufactured by Merck in Japan was utilized. In the automated HPLC system, both the extract and oleanolic acid standards, both in a volume of 10 µL, were injected. The column was kept at room temperature, and the entire separation was carried out in isocratic mobile phase with acetonitrile: water in the ratio (85:15) with a flow rate of 1.2 mL/min. Both the sample and the reference were measured at 348 nm. Oleanolic acid was detected through a comparison of extract retention time (Rt) to the standard [22].

### 2.9. In Vitro 11β-HSD1 Inhibitory Activity

The inhibitory action of 11β-HSD1 was assessed using fluorescence, but with some minor modifications. To perform the experiment, a 96-well plate was loaded with assay buffer (0.5 mM EDTA, 50 mM TrispH7.5, 150 mM NaCl, 200 mM NADP^+^, and 0.01% Brij35), dimethyl sulfoxide (DMSO) (0.00002%), and 10 μL of cortisol at various concentrations (0–200 mM). First, 170 μL of a 50 nM solution of 11-HSD1 in assay buffer was added to start the reactions, and then the mixture was incubated at 37 °C for 1 h. Finally, the reference medication STZ and extract were diluted in DMSO (0.00002%) and tested in assay buffer at 1 nM final concentration and 200 µL cortisol concentration. Finally, the reference drug (STZ) and extract were dissolved in DMSO (0.00002%) and evaluated in assay buffer at a final concentration of 1 nM and a cortisol concentration of 200 µL. After measuring the NADPH generation at 460 nm, the 11-beta-HSD1 activity was assessed [23].

### 2.10. Experimental Animals

At the Central Drug Research Institute in Lucknow, India, adult Swiss albino mice of either sex weighing between 35 and 40 g were purchased. They were kept in a 12 h dark/light cycle at room temperature (21 ± 2 °C) while fed rodent pellets and water ad libitum. Furthermore, the mice were kept in standard experimental conditions for a week to enable them to adjust. The Institutional Ethical Committee (IAEC) approved the protocols after reviewing them in accordance with the Committee’s recommendations (Approval No. 1213/ac/08/CPCSEA/IU).

### 2.11. Experimental Protocols

#### 2.11.1. Anti-Diabetic Activity in a Diabetes Model Induced by Streptozotocin (STZ)

Diabetes was induced in a total of 36 male Swiss albino mice, who were kept in acclimatization chambers for one week before the beginning of the research. The mice were then randomly separated into six groups, as follows:

Group I (normal control) gave a normal diet till the end of the experiment (28 days)
Group II (diabetes control) animals received 150 mg/kg BW streptozotocin
Group III (diabetes + Glibenclamide) received 5 mg/kg for 28 days
Group IV (diabetes + SMFE) received 100 mg/kg for 28 days
Group V (diabetes + SMFE) received 200 mg/kg for 28 days
Groups VI (diabetes + SMFE) received 400 mg/kg for 28 days

STZ-induced diabetes is characterized by beta-cell degeneration because of increased insulin levels in the blood, which are frequently absorbed by the cells. In experimental models, glibenclamide was used as a standard (positive control) to decrease blood glucose concentrations and increase insulin levels. Overnight-fasted mice were given 150 mg/kg of STZ monohydrate intraperitoneally dissolved in 0.9% *w*/*v* cold normal saline (12 h).

The mice were kept in their cages for 24 h to prevent hypoglycemia in 10% glucose solution bottles. Fasting blood glucose levels were measured 72 h after injection. Animals whose blood glucose levels remained below 200 mg/dL were excluded from the research. The 36 animals were split into two groups, one with diabetes and the other without. The first group (the controls) received 1 mL of distilled water via their feeding tubes to drink. Streptozotocin-induced diabetes in Group II was treated with 1 mL of distilled water daily morning, whereas streptozotocin-induced diabetes in Group III was treated with the reference medication glibenclamide (5 mg/kg/day). STZ was administered to the fourth, fifth, and sixth groups, who received 100, 200, and 400 mg/kg of SMFE daily. The extracts were administered to the animals once a day, before meals, via mandatory oral intubation. The treatment was continued for twenty-eight consecutive days. In addition, weekly measurements of overnight-fasted mice body weights and blood glucose levels were taken. At the end of the experiments, the animals were allowed to fast overnight before having blood drawn for biochemical analysis [24].

#### 2.11.2. Collection of Blood Samples for Glucose Analysis

After three days of STZ therapy (day 0), blood samples for glucose measurement were taken from overnight fasting mice through the tail veins method (12 h). These samples were obtained again on days 1, 7, 14, 21, and 28 after completing the STZ treatment. To assess glucose, the second drop of blood was added to an Accu-Chek glucose strip (Roche Diagnostics, Germany). In addition, ethanol was used as a disinfectant on the tails to lessen the likelihood of infection [25].

#### 2.11.3. Oral Glucose Tolerance Test (OGTT) in Mice

On the 15th day of treatment, both normal and drug-treated mice underwent an oral glucose tolerance test (OGTT) following a fasting interval of one night. A glucose load of 2 g/kg was administered orally to each mice using a feeding syringe precisely 30 min after the extract, standard medication, or vehicle had been delivered. Each mice blood glucose profile was taken before the glucose load (at time 0), after the glucose load (at times 30 and 60), and again at 120 min after the glucose injection. In the experiment, mice fasted for 18 h before receiving the glucose [26].

#### 2.11.4. Biochemical Parameters

Serum lipid levels were determined by taking blood samples from overnight fasting mice under diethyl ether anesthesia on days 0 and 28 using the tail vein procedure and were held aside for 30 min for clotting. Then, the sample was centrifuged at 3000 rpm for 10 min at 25 °C to separate the serum to determine the TC, TG, and HDL levels [27]. 

## 3. Result

**Figure 1:**
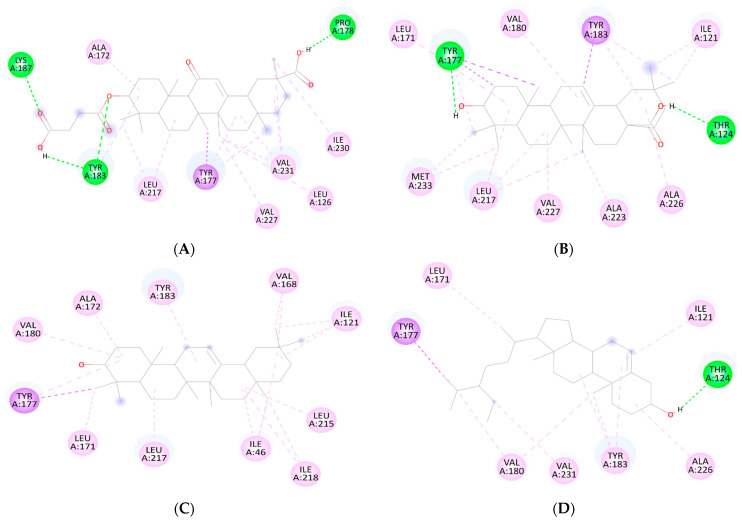
2D Binding orientation in the docked complex of the (**A**) carbenoxolone Acid, (**B**) oleanolic acid, β-amyrin (**C**), and β-sitosterol with 11-β-HSD1 (**D**). The compound is represented in green. The image was generated using the Discovery Studio Visualizer.

**Figure 2:**
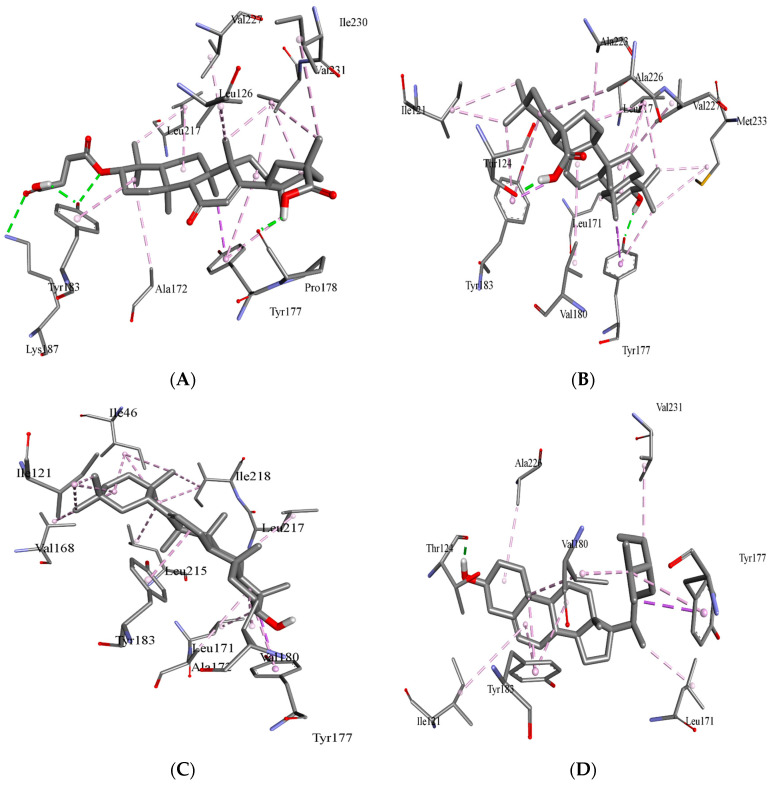
Binding orientation in the docked complex of the carbenoxolone acid (**A**) and oleanolic acid (**B**), β-sitosterol (**C**) and β-amyrin with 11 β-HSD1 (**D**). The image was generated using the Discovery Studio Visualizer.

Results of docking analyses for the anti-diabetic activity of detected compounds and standard carbenoxolone are shown in Table 1 and Figure 1 and Figure 2. All three compounds show binding energies (ranging between −11.11 and −11.57 kcal/mol), nearly similar to the standard (−11.50 kcal/mol).

### 3.1. Molecular Docking Analysis

The different conformations of carbenoxolone, β-sitosterol, β-amyrin, and oleanolic in the active site of 11-β-HSD1 are revealed by molecular docking simulation results. The highest binding energy is found in the best conformation. Figure 1 and Figure 2 depict the best binding pose of carbenoxolone, oleanolic acid, β-sitosterol, and β-amyrin. The best binding energy of complexes of oleanolic acid with 11-β-HSD1 was found to be −11.33 kcal/mol. In contrast, the docking score of docked carbenoxolone 11-β-HSD1 was found to be −11.9 kcal/mol. Remarkably, all compounds lack the hydrogen bond interaction with 11-β-HSD1, and the driving force for the binding is a network of hydrophobic bonds. Table 1 also shows the amino acid residues and the number of H-bonds involved in complex interaction.

### 3.2. Quantitative HPLC Analysis of Oleanolic Acid

**Figure 3:**
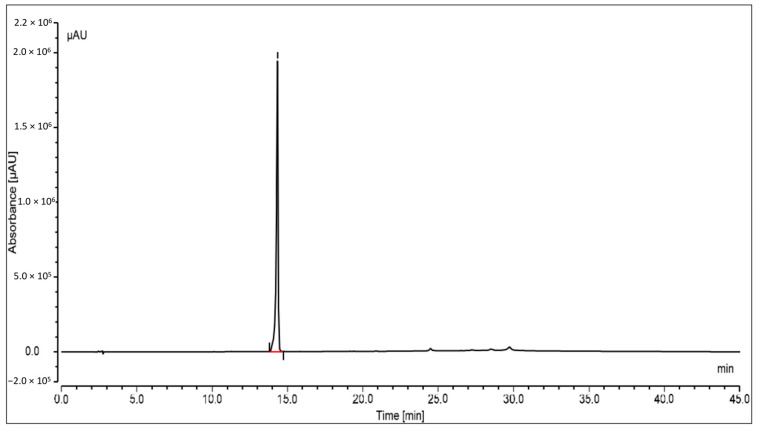
HPLC peak of standard oleanolic acid.

**Figure 4:**
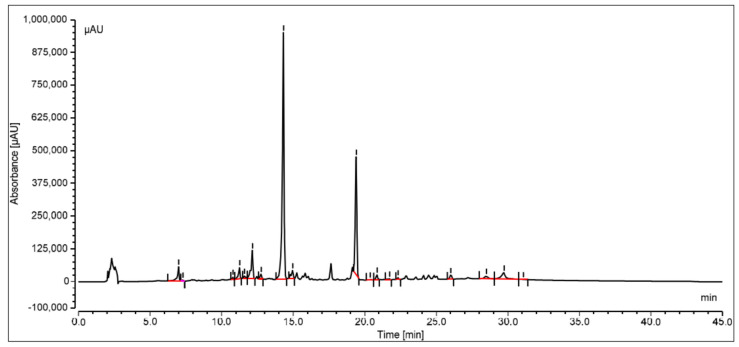
HPLC peak of oleanolic acid of *S. mangifera* fruit ethanolic fraction visualized at Rt 14.329.

The oleanolic acid in the extract was measured by reversed-phase high-performance liquid chromatography with a photo diode array (PDA) detector. At the stationary phase, an RP-C18 column was used, and at 348 nm, the compound was identified. Peak area versus concentration calibration curves plotted for oleanolic acid followed a straight line, as predicted by linear regression. At Rt14.329, a well-separated peak of oleanolic acid was observed. Oleanolic acid HPLC peaks for the standard and sample are displayed in Figure 3 and Figure 4, respectively. Oleanolic acid was measured at 1.31 ± 0.18% of the extract’s dry weight (*w*/*w*).

### 3.3. 11β. HSD1 Inhibitory Activity

**Figure 5:**
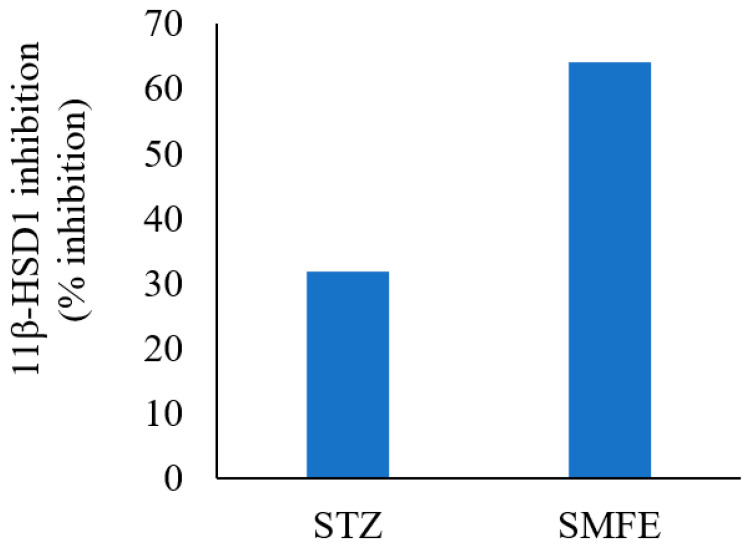
Effect of SMFE activity on 11β-HSD1 dehydrogenase activity (*n* = 3), *p* < 0.05.

The results of 11β HSD1 inhibitory assay shown in Figure 5 demonstrated that the extract of *S. mangifera* had a significant (*p* < 0.05) decrease in the 11-HSD1% inhibition (63.97%) in comparison to STZ (31.79%), which suggests that SMFE is a more effective inhibitory agent than the reference medication.

### 3.4. Effect of SMFE Extract in STZ-Induced Diabetic Mice

**Figure 6:**
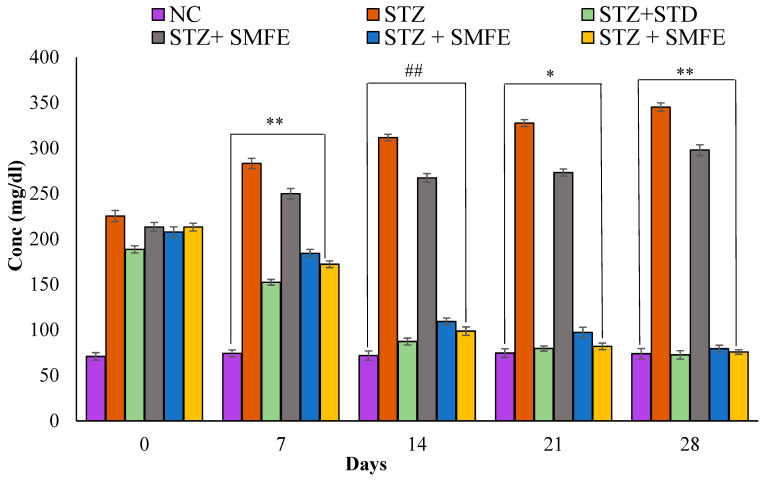
Analyzing the effects of SMF extract and glibenclamide on weekly blood glucose levels in both non-diabetic and STZ-induced diabetic mice. Data are represented as the mean ± SEM (*n* = 6). ** *p* < 0.01 = significant when compared with diabetic control. * *p* < 0.05 and ## *p* < 0.01 = significant when compared with diabetic control. NC = normal control; STZ = streptozotocin; SMFE = *S. mangifera* fruit extract; STD = standard drug (Glibenclamide).

In contrast to normal controls, STZ-induced diabetic mice exhibited significantly higher blood glucose levels (*p* < 0.01). Compared to the diabetic control group, the 200 mg/kg body weight and 400 mg/kg body weight doses of the SMFE extract caused significant (*p* < 0.05 and *p* < 0.01) reductions in blood glucose at 21 and 28 days, respectively. Additionally, when glibenclamide was given to group III at a 5 mg/kg dose, blood glucose levels dropped significantly (*p* < 0.01) on days 14, 21, and 28. Groups IV and V received SMF extract at doses of 200 and 400 mg/kg body weight, respectively, and showed a significant (*p* < 0.05 and 0.01) drop in blood glucose levels after 14, 21, and 28 days. On day 14, a maximum decrease in fasting BGL was observed (Figure 6).

### 3.5. Oral Glucose Tolerance Test (OGTT) in Mice

**Figure 7:**
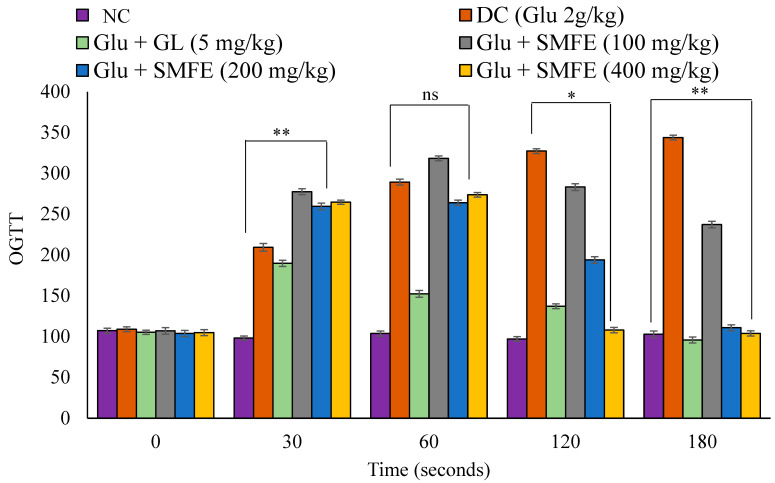
Effects of SMF extract on oral glucose tolerance test in mice. Data are represented as the mean ± SEM (*n* = 6). ** *p* < 0.01 = significant when compared with diabetic control group normal mice. * *p* < 0.05 = significant when compared with diabetic control. ns *p* > 0.05 = non-significant when compared with diabetic control. NC = normal control; SMFE = *S. mangifera* fruit extract; OGTT = oral glucose tolerance test; Gl = glibenclamide; Glu = glucose; DC = diabetes control.

During the oral glucose tolerance test, the experimental animals that had been given glucose considerably increased their blood glucose levels after 30 min. Regarding the animals in the disease control group, following a dose of 200 and 400 mg/kg of *S. mangifera* fruit extract, the blood glucose tolerance dropped significantly (*p* < 0.05 and *p* < 0.01) after 120 and 180 s. In contrast, the blood glucose levels of mice in group III that were given glibenclamide at a dose of 5 mg/kg exhibited substantial (*p* < 0.01) changes beginning at the 60 min mark (Figure 7).

### 3.6. Effect of SMFE on Lipid Parameters 

**Figure 8:**
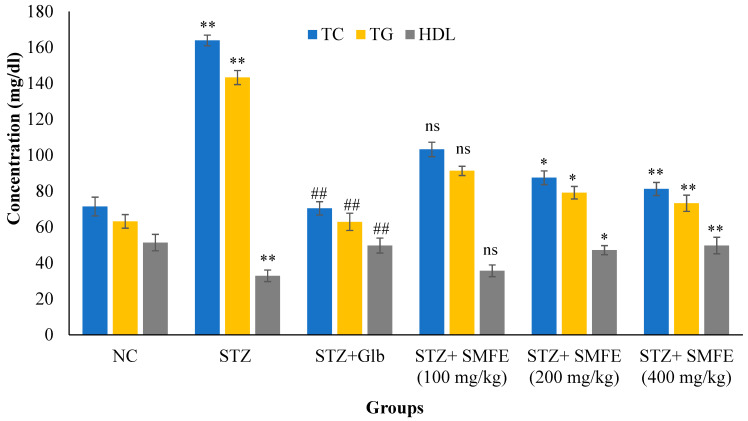
Effects of SMF extract on lipid parameters in mice after 28 days of therapy. Data are represented as the mean ± SEM (*n* = 6). Group I = normal control, Group II = toxic control, Group III = positive control, Group IV = ethanol extract 100 mg/kg, Group V = ethanol extract 200 mg/kg, Group VI = ethanol extract 400 mg/kg, ** *p* < 0.01 = significant when compared with the diabetic control group. * *p* < 0.05 and ## *p* < 0.01 = significant when compared with diabetic control. ns *p* > 0.05 = non-significant when compared with diabetic control. NC = normal control; STZ = streptozotocin; SMFE = *S. mangifera* fruit extract; Glb = glibenclamide.

Figure 8 shows the levels of total cholesterol and triglycerides of diabetic control animals were significantly (*p* < 0.01) increased, as well as the level of HDL-C significantly (*p* < 0.01) decreased in the same animals when compared with normal vehicle-treated group I animals. The administration of SMF extract in group IV, V, and VI experimental animals at a dose of 200 and 400 mg/kg significantly (*p* < 0.05 and 0.01) decreased the level of TC and TG and significantly increased (*p* < 0.01) the level of HDL-C when compared with group II animals. When 100 mg/kg of SMF extract was administered in group IV animals, they did not show significant effect (*p* > 0.05) on lipid parameters. 

### 3.7. Effect of SMFE on Body Weight in Mice

**Figure 9:**
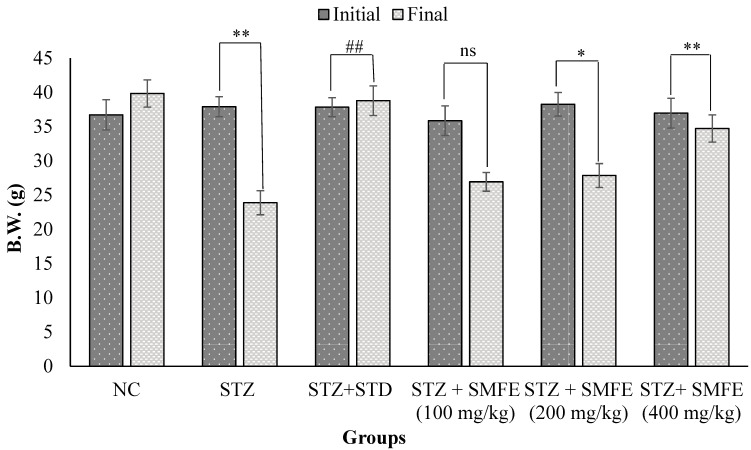
Effects of SMF extract and glibenclamide on body weight in mice. Data are represented as the mean ± SEM (*n* = 6). ** *p* < 0.01 = significant when compared with diabetic control group normal mice. * *p* < 0.05 and ## *p* < 0.01 = significant when compared with diabetic control. ns *p* > 0.05 = non-significant when compared with diabetic control. NC = normal control; STZ = streptozotocin; SMFE = *S. mangifera* fruit extract; STD = standard drug (glibenclamide).

Figure 9 illustrates the impact of SMFE extract on the body mass of diabetic mice. At the start of the study (0 days), there was no significant variation in body weight between the normal control group and all the experimental groups. However, in comparison to the diabetic control group, the SMFE extract 200 (*p* < 0.05), SMFE extract 400 (*p* < 0.01), and glibenclamide at a dosage of 5 mg/kg body weight all resulted in statistically significant (*p <* 0.01) weight increase at day 28 of therapy. On the other hand, those in the 100 mg/kg group IV mice did not exhibit any signs of weight increase.

## 4. Discussion

Though there are effective pharmaceutical treatments for diabetes, the ethnobotanical community emphasizes herbal therapies and extracts, believing they are safer alternatives than synthetic drugs. Traditional and herbal medicines have aroused the scientific community’s attention due to their pharmacological and economic advantages [28]. Furthermore, the drug’s development may benefit significantly from using natural products since they provide a broad and abundant supply of compounds. Such compounds’ structural and chemical diversity has led to several important discoveries [29]. *S. mangifera* is a popular plant. The fruits and aerial parts contain a variety of phytoconstituents, such as daucosterol, cycloartanone-24-methylene, β-sitosterol, β-amyrin, lignoceric acid, dausterol, and oleanolic acid. In addition, it has fewer beneficial compounds such as galloylgeranin, alanine, niacin, and riboflavin [21]. The current examination was conducted to assess the anti-diabetic properties of the ethanolic fruit extract of *S. mangifera* by in silico, in vitro, and in vivo studies.

11β-hydroxysteroid dehydrogenase type 1 (11β-HSD1) is a homodimer enzyme responsible for reducing cortisone to its active form, cortisol [30]. Overexpression of 11β-HSD 1 in key metabolic tissues can lead to metabolic changes such as insulin resistance, hyperglycemia, and type 2 diabetes [31]. The inhibition of 11β-HSD1 has been shown to attenuate the effect of the previous metabolic changes and other diseases mediated by excessive cortisol production. Therefore, 11β-HSD1 inhibition may offer a new therapeutic approach for type 2 diabetes mellitus. 11β-HSD1 active site consists of highly conserved residues (SER170, TYR177, VAL180, and TYR183) in addition to LEU217 and TYR284, which stabilize the C-terminal loop of the protein during interaction [32,33].

β-sitosterol scored the lowest binding energy (−11.57 kcal/mol), which binds to the enzyme active site through one hydrogen bond with THR124—as oleanolic acid—and seven hydrophobic interactions (π–π stacking and π–σ interactions) with ILE121, LEU171, TYR177, VAL180, TYR183, ALA226, and VAL231. β-amyrin (−11.32 kcal/mol) and oleanolic acid (−11.11 kcal/mol) share the same binding behavior to the active site (ILE121, LEU171, TYR177, VAL180, TYR183, and LEU217) and that is due to the high structural similarity between them. It is important to note that residues TYR183, TYR177, and VAL180 are involved in the binding of carbenoxolone, and all three compounds’ techniques have been utilized to examine possible interactions between prospective targets 11-β-HSD1 and compounds produced from natural products, such as β-sitosterol, β-amyrin, and oleanolic acid, heading to the invention of new pharmacological frontiers. In silico results clearly showed the best binding energy of complexes of oleanolic acid with 11-β-HSD1 was found to be −11.11 kcal/mol. Using computational molecular docking, we found that oleanolic acid and β-amyrin interacted with six amino acid residues of targets 11-β-HSD1, providing insight into the molecular binding mechanism of 11-β-HSD1 in the catalytic region (Figure 1 and Figure 2, and Table 1). In silico study of the phytoconstituents of *S. mangifera* on 11β-HSD1 found that all tested compounds had shown good docking scores and significant interactions with the catalytic region, consenting them to block the enzyme. 

The therapeutic potential of oleanolic acid, a naturally occurring component of many plant-based foods and medicines, has the potential to directly affect enzymes involved in insulin production, secretion, and signaling [34]. In the current study based on the in-silico results, the optimal binding energy of oleanolic acid complexes with 11-β-HSD1 was determined to be −11.11 kcal/mol. On the other hand, the docking score of docked carbenoxolone 11-β-HSD1 molecule was found to be −11.9 kcal/mol. Additionally, the SMFE extract oleanolic acid concentration was analyzed using reversed-phase HPLC and a photo diode array (PDA) detector. Oleanolic acid constituted 1.31 ± 0.18% of the extract’s dry weight with a clear peak at Rt14.329. The findings of the in vitro 11β HSD1 inhibitory experiment revealed that the extract of *S. mangifera* had a significant (*p* < 0.05) reduction in the 11-HSD1% inhibition (63.97%) compared to STZ (31.79%), indicating that SMFE is a more effective inhibitory agent than the reference medicine. It was reported that therapy with oleanolic acid improved insulin sensitivity by increasing the expression of insulin receptor and glucose transporter proteins in HepG2 cells, which is significant because insulin resistance is a hallmark of type 2 DM [35]. 

Analyzing the effects of glibenclamide and SMF extract on blood glucose levels as evaluated weekly in both STZ-induced diabetic and normal mice showed the putative anti-diabetic benefits of SMFE to those of glibenclamide. The primary function of glibenclamide is to stimulate insulin secretion; this is the mechanism by which it works [36]. It is well established that both STZ-induced diabetic and normal mice experience hypoglycemia following receiving an oral dosage of hypoglycemic medications [37]. In addition, it has also been discovered that glibenclamide is effective in animals that are diabetic to a moderate degree but that it is not helpful in animals that are diabetic to an extreme degree, in which the pancreatic beta cells have been destroyed almost entirely [38]. Therefore, the anti-hyperglycemic benefits of several plant extracts have been hypothesized to originate from their capability to stimulate the progression of new β-cells, prevent the death of pancreatic cells, reduce glucose load, or increase the free flow of the body’s insulin. Additionally, they could cause β-cells to secrete insulin or turn on insulin receptors. It has been shown that some plants have glucose-lowering effects on normal glycemic animals comparable to those of the popular sulfonylurea medication gliclazide [39]. Through the production of alkylating free radicals, STZ administration results in the necrosis of β-cells. 

Most diabetic problems in all individuals are caused by persistent hyperglycemia, a frequent feature of diabetes. As a result, treatment should focus on carrying blood glucose levels low to within normal values [40]. In the current study, treatment of SMFE at doses of 200 and 400 mg/kg was shown to have an apparent anti-hyperglycemic effect and a significant reduction in blood glucose levels at the 14th, 21st, and 28th days (*p* < 0.05 and 0.01). This may be because it increases insulin release from pre-existing β-cells in the body. In addition, abnormally low body weight can recognize STZ-induced diabetes because it causes the breakup of structural proteins known to contribute to overall body weight [41]. Compared to diabetic control mice, SMF extract (200 and 400 mg/kg)-treated diabetic animals did not lose body weight, demonstrating the SMFE potential to lower hyperglycemia. However, at the end of the treatment (4 weeks), the diabetic animals lost weight. This is shown in Figure 8, which shows the bodyweight changes and biochemical variables of the various groups of experimental animals. Compared to non-diabetic animals, the untreated diabetes group had constant weight loss. Though, compared to the diabetic control group, the SMF extract-treated group for four weeks at doses of 200 and 400 mg/kg body weight exhibited a very significant (*p* < 0.01 and 0.05) rise in body weight.

In addition, glucose-loaded mice that were given SMF extract orally at dosages of 200 mg/kg and 400 mg/kg exhibited a substantial decrease (*p* < 0.05 and 0.01) in BGL at 120 and 180 min after treatment when compared to glucose-loaded untreated groups. Additionally, glibenclamide has shown a considerable reduction beginning at the 30 min mark. This suggests that the extract can enhance glucose tolerance in normal mice, which may have implications for reducing the postprandial hypoglycemia linked with diabetes. Controlling postprandial hypoglycemia is one of the techniques for managing type 2 diabetes, and OGTT is a test that measures the body’s capacity to use glucose and is used as a routine procedure in clinical settings to diagnose borderline diabetic individuals [42]. The capacity to improve glucose tolerance may result from several plausible mechanisms, such as stimulation of glycogenesis in the liver, increased tissue glucose consumption, and reduced gluconeogenesis [43]. 

When the serum glucose level is below ≤60 mg/dL, physiological responses are induced. These physiological responses include the release of cortisol, epinephrine, growth hormone, and glucagon, which can help adjust serum glucose levels. These responses work together to stimulate gluconeogenesis, glycogenolysis, lipolysis, and protein breakdown. This effect might be obscured if the plant extract being studied does seem to have any hypoglycemic influence in either normal mice or diabetic mice. In order to avoid this confounding variable, we examined the effect of SMFE after giving glucose loads to either normal or diabetic mice. Hyperglycemia makes people lose weight because they eat more fats and structural proteins than carbs [44], and carbohydrates are used and controlled by insulin [45]. During this research, the diabetic control group considerably decreased body weight. Animals in group II that were given glibenclamide at a dose of 5 mg/kg experienced significant improvements in their body weights compared to the diabetic control group. The reversal of processes such as gluconeogenesis, glycogenolysis, and proteolysis may be responsible for the restoration of weight [46].

The impact of alcoholic extracts of SMFE on lowering hyperlipidemia in mice with STZ-induced diabetes showed an increase in TC and TG levels after 28 days of therapy in the diabetic control group, whereas decreases were seen in HDL. Due to the inefficient use of glucose, diabetes-related hyperlipidemia is caused by an increase in fat mobilization from adipose tissue [47]. More fatty acids are released into the bloodstream from stored triacylglycerols in a diabetic condition because hormone-sensitive lipase is more efficient at converting them. Because of this, the liver is stimulated to produce more phospholipids and cholesterol from its surplus of fatty acids. Lipoproteins are created when phospholipids, cholesterol, and excess TG are released into the bloodstream [48].

Additionally, the diabetes condition makes the triglyceride-hydrolyzing enzyme lipoprotein lipase ineffective. As a direct consequence, hypertriglyceridemia and a drop in HDL values might be seen [49]. Furthermore, hypercholesterolemia may occur when a person has diabetes because insulin inhibits β-hydroxy-β-methylglutaryl coenzyme-A (HMG-CoA) reductase, a major rate-limiting enzyme called in the degradation of cholesterol-rich LDL components [50]. As a result, total cholesterol and triglyceride levels decreased considerably (*p* < 0.05) in the *S. mangifera* diabetic positive control groups. In contrast, HDL levels were significantly enhanced (*p* < 0.05). In addition, by day 14, TC and TG levels were within normal ranges for all *S. mangifera* diabetic positive control groups.

In contrast, HDL levels were elevated to the normal range for the 400 mg/kg *S. mangifera* diabetic positive control group. This might be because enhanced glucose consumption (as shown by a decline in glucose levels) led to the prevention of lipid peroxidation and regulation of lipolytic hormones. Various plants have been documented in this approach to have anti-hyperlipidemic properties [51]. In addition, multiple saponins derived from plants have been shown in other investigations to have considerable anti-hyperlipidemic actions, mainly via the reduction of cholesterol luminal absorption and the enhancement of cholesterol production through biliary excretion [52,53]. 

Diabetes is associated with an increased risk of atherosclerosis and cardiovascular disease due to high triglycerides, cholesterol, and low-density lipoprotein (LDL) [54]. As a result, reducing blood cholesterol levels while increasing HDL by dietary or drug treatment appears to be linked with a decreased risk of cardiovascular disease and associated consequences [48]. This is the first research showing that *S. mangifera* may lower blood sugar and cholesterol levels. This highlights the need for additional research on *S. mangifera* since many anti-hyperglycemic medicines only reduce blood glucose concentrations without adequately controlling hyperlipidemia.

## 5. Conclusions

The incidence of type 2 diabetes has exploded because of demographic shifts toward an increasingly older and less active population and a rise in rates of overweight and obesity. Thus, novel therapeutic targets and anti-diabetic drugs with reduced side effects and enhanced efficacy are required for the prevention and management of T2DM. Naturally occurring triterpenoids found in functional foods present an intriguing alternate option. Findings from enzymatic and molecular docking investigations showed that the primary bioactive compounds of *S. mangifera* (β-amyrin, β-sitosterol, and oleanolic acid) exhibited sub-chronic and acute anti-diabetic impact through enhancing lipids and glucose metabolism, which may have been mediated by 11β-HSD1 inhibition. Additionally, HPLC analysis of SMFE was performed for the presence of oleanolic acid, which was shown to be present at 1.31 ± 0.18% of the extract’s dry weight (*w*/*w*). Furthermore, in vivo tests validated that SMFE had an anti-hyperglycemic impact by reducing glucose absorption. In silico, in vitro, and in vivo investigations suggest that SMFE have shown prolonged anti-diabetic and anti-hyperglycemic effects, which may be interceded by an insulin sensitization with subsequent modifications of triglycerides, cholesterol, and glucose. In conclusion, we suggest *S. mangifera* fruits as potential anti-diabetic medicines and hit structures for developing more effective and targeted pharmaceuticals.

## Figures and Tables

**Table 1:** Summary of selected constituents and reference compounds (carbenoxolone). Chemical structures Autodock docking scores, hydrogen bond interactions, and hydrophobic interaction residues.

S.No.	Compound	Minimum Binding Energy on 11β-HSD1 (PDB ID-2ILT) (kcal/mol)	Interaction (H-Bond)	Interaction (Hydrophobic)
1.	β-Sitosterol	−11.57	Thr A 124	LeuA: 171, TyrA: 177, IleA: 121, ValA: 180, ValA: 231, TyrA: 183, AlaA: 226
2.	β-Amyrin	−11.32	--	ValA: 180, AlaA: 172, TyrA: 183, ValA: 168, IleA: 121, TyrA: 177, LeuA: 171, LeuA: 217, IleA: 46, IleA: 218, LeuA: 215
3.	Oleanolic acid	−11.11	Tyr A177 Thr A 124	LeuA: 171, ValA: 180, TyrA: 183, IleA: 121, MetA: 233, LeuA217, ValA: 227, AlaA: 223, AlaA: 226
4.	Carbenoxolone	−11.50	Lys A18 Pro A:178 Tyr A 183	Ala A: 172, Leu A: 217, Tyr A: 177, Val A: 227, Val A: 231, Leu A:126, Ile A:230

## Data Availability

The data presented in this study are available on request from the corresponding authors.
